# Neural Conversational Agent for Weight Loss Counseling: Protocol for an Implementation and Feasibility Study

**DOI:** 10.2196/60361

**Published:** 2024-09-20

**Authors:** Alexander Kotov, April Idalski Carcone, Elizabeth Towner

**Affiliations:** 1 Department of Computer Science College of Engineering Wayne State University Detroit, MI United States; 2 Department of Family Medicine and Public Health Sciences School of Medicine Wayne State University Detroit, MI United States

**Keywords:** conversational agents, artificial intelligence, behavior change, weight loss, obesity, motivational interviewing, web-based application, deep learning, transformers, large language models, feasibility study

## Abstract

**Background:**

Obesity is a common, serious and costly chronic disease. Current clinical practice guidelines recommend that providers augment the longitudinal care of people living with obesity with consistent support for the development of self-efficacy and motivation to modify their lifestyle behaviors. Lifestyle behavior change aligns with the goals of motivational interviewing (MI), a client-centered yet directive counseling modality. However, training health care providers to be proficient in MI is expensive and time-consuming, resulting in a lack of trained counselors and limiting the widespread adoption of MI in clinical practice. Artificial intelligence (AI) counselors accessible via the internet can help circumvent these barriers.

**Objective:**

The primary objective is to explore the feasibility of conducting unscripted MI-consistent counseling using Neural Agent for Obesity Motivational Interviewing (NAOMI), a large language model (LLM)–based web app for weight loss counseling. The secondary objectives are to test the acceptability and usability of NAOMI’s counseling and examine its ability to shift motivational precursors in a sample of patients with overweight and obesity recruited from primary care clinics.

**Methods:**

NAOMI will be developed based on recent advances in deep learning in four stages. In stages 1 and 2, NAOMI will be implemented using an open-source foundation LLM and (1) few-shot learning based on a prompt with task-specific instructions and (2) domain adaptation strategy based on fine-tuning LLM using a large corpus of general psychotherapy and MI treatment transcripts. In stages 3 and 4, we will refine the best of these 2 approaches. Each NAOMI version will be evaluated using a mixed methods approach in which 10 adults (18-65 years) meeting the criteria for overweight or obesity (25.0≥BMI≤39.9) interact with NAOMI and provide feedback. NAOMI’s fidelity to the MI framework will be assessed using the Motivational Interviewing Treatment Integrity scale. Participants’ general perceptions of AI conversational agents and NAOMI specifically will be assessed via Pre- and Post-Interaction Questionnaires. Motivational precursors, such as participants’ confidence, importance, and readiness for changing lifestyle behaviors (eg, diet and activity), will be measured before and after the interaction, and 1 week later. A qualitative analysis of changes in the measures of perceptions of AI agents and counselors and motivational precursors will be performed. Participants will rate NAOMI’s usability and empathic skills post interaction via questionnaire-based assessments along with providing feedback about their experience with NAOMI via a qualitative interview.

**Results:**

NAOMI (version 1.0) has been developed. Participant recruitment will commence in September 2024. Data collection activities are expected to conclude in May 2025.

**Conclusions:**

If proven effective, LLM-based counseling agents can become a cost-effective approach for addressing the obesity epidemic at a public health level. They can also have a broad, transformative impact on the delivery of MI and other psychotherapeutic treatment modalities extending their reach and broadening access.

**International Registered Report Identifier (IRRID):**

PRR1-10.2196/60361

## Introduction

### Background

Obesity, defined as having a BMI exceeding 30 kg/m^2^ is a complex chronic disease, in which abnormal or excess adiposity (ie, body fat) impairs health [1]. It is a common, serious, and costly disease. A recent nationally representative survey [2] found that nearly 1 in 3 (30.7%) US adults are overweight (have a BMI in the range of 25-29.9 kg/m^2^), more than 2 in 5 (42.4%) are obese, and about 1 in 11 (9.2%) have severe obesity (BMI exceeding 39.9 kg/m^2^). Obesity is strongly correlated with increased morbidity and mortality [3], reduced lifespan [1], and an increased risk of metabolic, cardiovascular, musculoskeletal, and psychiatric diseases and conditions [4]. Individuals with obesity incur significantly higher medical costs than individuals without obesity, both overall and for most major categories of health expenditures [5]. The annual cost of adult obesity in the US including direct costs of treating obesity-related diseases and conditions, lost wages, short-/long-term disability, and lost productivity is estimated to exceed US $200 billion [6]. Additionally, people with obesity experience pervasive weight bias and stigma, which further contribute to increased morbidity and mortality [7]. Despite significant attention and funding [8-10], obesity rates in both adults and youth in the United States and worldwide continue to increase [11-13], which poses a significant public health problem.

The most common cause of obesity is a chronic caloric imbalance [14] attributed to lifestyle factors, such as diet and physical activity [15]. Depending on severity, the treatment options for obesity range from psychological interventions and lifestyle modification to pharmacotherapy and surgery. Current clinical practice guidelines [1] suggest incorporating multicomponent psychological interventions combining behavior modification (goal setting, self-monitoring, and problem-solving), cognitive reframing, and value-based strategies to alter diet and activity levels into all obesity treatment plans. Moreover, health care providers are advised to augment longitudinal care of people living with obesity with consistent messaging to support the development of self-efficacy (confidence in one’s abilities to enact behavior changes) and intrinsic motivation (desire to engage in behavior changes for reasons of personal interest or satisfaction) for lifestyle modifications supporting weight loss. Such messaging aligns with motivational interviewing (MI) [16,17], a client-centered, yet directive counseling modality aiming to enhance self-efficacy and intrinsic motivation toward behavior change [18,19]. Originally developed to address addiction [20], alcohol [21-23], and substance abuse [24,25], MI has a growing evidence base for health behavior change [26], including smoking cessation [27], HIV medication adherence [28], as well as the treatment of depression, anxiety, and other mental health conditions [29-35].

The widespread adoption of MI into primary care faces 2 major barriers. First, training health care providers to be proficient in MI is expensive and time-consuming [36] and the typical training approaches have a modest, diminishing effect on MI fidelity [37,38]. This often results in modest outcomes (anthropometric changes in the case of obesity [39]). Second, there is a shortage of qualified mental and behavioral health counselors [40]. Artificial intelligence (AI) counselors that are accessible via the internet to deliver MI-based weight loss counseling can help mitigate these barriers as well as extend the reach and broaden access to weight loss counseling beyond primary care. Accessible 24/7 in any location, these counselors can provide in-the-moment support during times of high need (eg, social situations). They can complement human counselors in the health care delivery continuum by delivering weight loss interventions to patients with mild- and early-stage obesity, supporting in-person counseling between visits, and via preventive interventions to patients at risk for developing obesity. This approach has the potential to conserve costly therapeutic resources for those with more severe obesity and less responsive to treatment. Another option is to leverage AI counselors as part of a hybrid strategy to boost motivation before initiating more intensive in-person weight loss interventions.

Although automated counselors in the form of embodied agents [41-45], health coaches [46,47], and patient advisors [48,49] featuring animated human-like characters with emulated speech, gaze, posture, and gestures are visually appealing, their ability to engage the patients in a natural dialog is severely limited due to their reliance on scripted interactions, in which patients must choose from a set of actions (eg, responses, requests, and questions) predefined for each situation anticipated during an intervention. The responses of these counselors are also either completely predefined or templatized, which further constrains patient interaction and limits the scope and effectiveness of behavioral and mental health interventions involving free-form communication [50], such as MI counseling [16,51].

Recent advances in deep learning [52], including the emergence of the Transformer architecture [53] and large language models (LLMs) [54], which excel at question answering, text generation, and summarization, have enabled AI-based conversational agents to engage in nontemplatized, unscripted, human-like dialogue. These advances have paved the way for testing the feasibility of using deep learning–based conversational AI agents to deliver free-form (ie, unscripted) MI-style weight loss counseling. However, no studies of fully automated deep learning–based generative AI counselors have been reported in the literature.

### Goals of This Study

The main objectives of this study are 2-fold. The primary objective is to explore the feasibility of conducting unscripted weight loss MI with a fully automated LLM-based AI counselor by developing Neural Agent for Obesity Motivational Interviewing (NAOMI), a Web app for MI-style weight loss counseling. The secondary objectives are to test the acceptability and usability of NAOMI’s counseling and examine its ability to shift motivational precursors in a sample of overweight and obese patients recruited from primary health clinics.

Because this is a feasibility study involving a single interaction with NAOMI, it is not expected that participants will experience anthropometric changes. Participants will be advised to continue their current weight loss interventions (if any) and those who wish to engage in a weight loss intervention will be encouraged to contact their primary care provider for a referral to a weight loss treatment.

### Prior Work

#### Overview

This study builds on recent advances across 3 research areas: dialog systems, Transformer-based neural architectures and LLMs; conversational agents for mental health care; and natural language processing methods for annotating MI session transcripts. Below we provide a detailed overview of prior work in each area.

#### Dialog Systems, Transformers, and LLMs

General-purpose dialog systems are traditionally categorized [55] into goal-oriented [56] and non–goal-oriented [57]. Non–goal-oriented dialogue systems, commonly referred to as chatbots, try to maintain a human-like “small talk” or casual conversation without any specific goal and for as long as possible. Goal-oriented dialog systems, on the other hand, explicitly incorporate a supervision signal, such as progress towards completion of a certain task (eg, reserving a table at a restaurant). This type of dialog system structures human-computer communication as a sequence of distinct dialog states*,* with each dialog state defined in terms of values of discrete variables (ie, slots, such as location, time, party size, and type of cuisine for restaurant reservations). Goal-oriented dialog systems (eg, intelligent assistants) are also typically integrated with automated schedulers or databases and maintain a conversation with their users until all the information necessary to accomplish a task has been collected. Conversational agents for behavioral counseling fall into the category of specialized dialog systems. They are different from general-purpose dialog systems and other conversational agents in health and medical care [58], since they need to effectively use a variety of counselor communication skills at appropriate moments during a loosely structured and time-limited interaction with users.

The advent of the Transformer [53], an encoder-decoder neural architecture with a multihead attention mechanism, has revolutionized machine learning, including natural language processing and dialog systems. Neural models based on the Transformer architecture are typically pretrained on large human-written textual corpora consisting of tokens (words, punctuation marks, emojis, etc) to minimize the error on several computational tasks, most commonly to predict a token given a sequence of tokens (the context). The Transformer gave rise to 3 types of neural architectures. Architectures of the first type, which include Bidirectional Encoder Representations from Transformers [59] and its variants [60-63], use only the encoder stack of the Transformer and are typically used in a transfer learning scenario to create dense representations of text for a particular downstream natural language processing task. Specifically, they are pretrained on a large textual corpus using the token prediction and next-sentence classification objectives and fine-tuned on a much smaller corpus for a specific downstream task, such as text classification. The architectures of the second type, which include BART [64] and T5 [65], use the encoder and decoder stacks of the Transformer and are typically used for text summarization. Models of the third type, which include foundation LLMs [54], such as GPT [66], ChatGPT [67], PaLM [68], and LLaMa [69], use only the decoder stack of the Transformer architecture. Rather than being specialized architectures developed for a particular task or for creating representations that can be fine-tuned for a certain task, foundation LLMs are trained to attain general-purpose cognitive capabilities and help users accomplish many different tasks. Such LLMs are typically used in zero-shot or few-shot learning scenarios when they are provided with a textual prompt describing a problem optionally accompanied by several examples of inputs and solutions [70].

In the context of health care, the Bidirectional Encoder Representations from Transformers architecture trained on clinical notes was shown to accurately predict patients’ readmission, in-hospital mortality, comorbidity index, length of stay, and insurance denial [71]. Other studies [72,73] found that foundation LLMs possess significant medical knowledge and can write medical notes based on transcripts of physician-provider encounters, solve problems from board examination with accuracy similar to or surpassing human physicians (with the highest scores in psychiatry), or interactively provide a “curbside consult” given a clinical case summary. Foundation and other Transformer-based LLMs can also be used in dialog systems to generate system responses given the context of prior interactions with users. Due to the central role language and conversation play in the description, manifestation, and treatment of mental health disorders, LLMs hold marked potential for mental health care and psychiatry [74]. Neural architectures (including the Transformer-based ones) have been previously adapted to generate specific counselor language, such as empathy (statements of support and understanding of the client’s experiences and emotions) [75-77] and reflections (repetitions or rephrasing of the client’s prior statements) [78,79]. Partner [80], a system combining reinforcement learning and GPT-2, was proposed and evaluated for empathic rewriting of responses in online mental health support platforms.

#### Conversational Agents for Mental Health Care

The use of conversational agents [58] and LLMs [81] in health care, including mental health care and psychiatry [82], is increasing in frequency. Recent systematic reviews [83-85] established that conversational agents have been tested in the delivery of mental health services, most commonly focused on providing support during psychological distress (such as dealing with stress, depression, anxiety, or posttraumatic stress disorder), health behavior counseling and promoting psychological well-being, with cognitive behavioral therapy most frequently used as psychotherapeutic modality. The vast majority of existing conversational agents for mental health care, promoting psychological well-being, and health behavior counseling are rule- or retrieval-based [86]. Consequently, such agents require a substantial amount of manual input in the form of dialog scripts, action rules, and agent response databases. By directing conversations through a set of predefined or templatized responses, such agents are prone to unnatural, impersonal, or repetitive communication, which reduces their clinical effectiveness [86]. For example, Bonobot [87], a nonneural text messaging conversational agent for MI on stress management, uses predefined session scripts and templates triggered by the keywords in patient utterances to generate responses. It is built on top of ELIZA [88], a system developed more than half a century ago to mimic a Rogerian psychotherapist by persistently rephrasing patient statements and asking questions. Woebot (Woebot Health), [89] a mobile phone app for mood tracking and delivering cognitive behavior therapy, permits only constrained user input (eg, a set of utterances predefined for each dialog situation) and responds with predefined counselor phrases. Lark [90], a mobile health coach, responds to users’ specific input, such as food and beverage consumption, weight, or sleep duration, with predefined content (eg, praise or educational material). Embodied conversational agents [91] focus on the visual modality of automated behavioral health interventions, restricting user input in a scripted dialog to selecting predefined answers to the agent’s templatized questions [41,44,45,92,93].

While neural conversational agents have been previously developed to simulate patients for training human counselors [94], we are unaware of any previously published research on fully automated neural generative (ie, using neural architectures to generate from scratch rather than retrieve predefined responses) conversational agents for MI-style counseling. Therefore, whether it is feasible to use such agents for delivering free-form MI interventions remains an open research question. The recent paper by Brown et al [95], reporting the results of an iterative study of an MI agent for smoking cessation is the closest prior work to this study. However, the agent evaluated in this study is significantly simpler and only generates reflections of the users’ responses in a scripted dialog consisting of just 5 predefined open-ended questions.

#### Natural Language Processing Methods for MI Session Transcripts

Previously proposed machine and deep learning methods for the analysis of MI session transcripts fall into 2 categories: methods for identification and characterization of counselor communication behaviors and methods for session-level analysis and prediction.

##### Identification and Characterization of Counselor Communication Behaviors

The methods in this category include machine [96-98] and deep learning [97-101] binary classifiers to identify counselor communication behaviors, such as expressions of empathy [102,103] and multiclass classifiers [96-98,100,101,104] to identify specific or a range of counselor or client communication behaviors. Such classifiers are primarily used for auto-coding MI transcripts, a common first step in a retrospective analysis of MI session transcripts to identify effective and ineffective counselor communication patterns. Other prior work in this category focused on identifying acoustic [102] and contextual [103] markers of empathy, extracting linguistic patterns associated with specific counselor communication behaviors [105], predicting counselor [105] or client [100] communication behaviors, and automated scoring of human MI counselor reflections [106].

##### Session-Level Analysis and Prediction

The methods in this category take MI session transcripts manually or automatically annotated with behavior codes as input to predict the likelihood of eliciting target patient language [107] and identify effective counselor communication strategies [108]. Other methods in this category include a method to discover the discourse structure in email-based MI sessions [109] and a method to discover linguistic differences between high-quality and low-quality MI sessions [110].

### Study Hypotheses

This study will explore the following hypotheses: (1) NAOMI will achieve at least intermediate MI fidelity based on Motivational Interviewing Treatment Integrity (MITI) coding scheme [111] (see the Statistical Analysis subsection of the Methods section for more details); (2) At least 80% of the last cohort of study participants will find NAOMI acceptable and usable; specifically, they will like using NAOMI, be comfortable discussing their weight with it, find their interaction with it pleasant, its guidance helpful, and want to use it again (see the Measures subsection of the Methods section for more details); (3) Interaction with NAOMI will result in positive shifts in all motivational precursors (see the Qualitative Analysis subsection of the Methods section for more details).

## Methods

In this section, we provide the details of the overall study design, recruitment activities, and measures that will be used to assess the acceptability and usability of NAOMI and explore change in motivational precursors.

### Overview

The design and implementation of this research protocol have three main strengths: (1) a strong multidisciplinary team consisting of computer scientists and behavioral health researchers with specific expertise in social work, clinical psychology, intervention development, and health equity; (2) partnerships with primary care clinics interested in providing evidence-based care and eliminating health disparities; and (3) the methods and techniques feasibility tested in this project can, in principle, be adapted to a wide spectrum of psychotherapeutic interventions.

### Study Design

#### Overview

We are planning 4 stages of NAOMI’s development and validation with each stage following an explanatory mixed methods design [112]. The results of stages 1 and 2 will inform development activities in the remaining stages. Study participants will complete an in-person or online study visit and a follow-up survey 1 week later.

#### Study Visit

Participants will complete an in-person study visit using a tablet computer. If a study visit takes place online, the participants will complete the visit on a device of their choice using Zoom. The structure of the study visit is detailed in Textbox 1.

**Structure of a study visit.**

Prior to the commencement of study visit activities, study staff will provide and review a copy of the study information sheet with eligible and interested patients and allow prospective participants to ask any questions. Prospective participants will be informed that by proceeding with study activities, they are consenting to participate in the study, however, their participation is completely voluntary and can stop at any time. Upon verbal acknowledgment from the prospective participant, study activities will proceed as follows:

1. Study participants will complete the Participant Information Form and the Pre-Interaction Questionnaire (described below).
2. Participants will then interact with Neural Agent for Obesity Motivational Interviewing (NAOMI) using a tablet computer (with a detachable keyboard) for at least 10 minutes but no more than 1 hour.
3. Participants will complete the Post-Interaction Questionnaire, Usability Questionnaire, and the Consultation and Relational Empathy Measure (described below).
4. Participants will complete a Semi-Structured Qualitative Interview to share their impressions of NAOMI and suggestions for its improvement.

#### Follow-Up

One week after the study visit, participants will be sent a link to the online Follow-Up Questionnaire (described below).

### Measures

The study measures described below will be administered online using Qualtrics.

#### Participant Information Form

The study investigators developed this form to collect demographic information including the participant’s age, sex, gender identity, racial/ethnic background, marital status, education, employment status, income levels, and perceptions of their weight and food security. Completing this form should take no more than 10 minutes.

#### Pre-Interaction Questionnaire

This 15-question instrument includes 12 five-point Likert-scale questions adapted from the Psychometric Analysis of the Perceptions of Computerized Therapy Questionnaire-Patient version (PCTQ-P) [113]. The PCTQ-P is a validated measure grounded in the diffusion of innovations theory [114] previously shown to be effective at predicting successful dissemination of evidence-based mental health interventions [115]. The goal of these questions is to assess the participants’ degree of exposure to and perceptions of AI conversational agents in general, including the detection of any presuppositions and biases towards AI, their attitude about AI counselors, preference for AI over human counselors, and belief in the ability of AI counselors to facilitate weight loss. The questions about participants’ attitudes toward AI counselors, preference for AI over human counselors, and beliefs in the ability of AI counselors to facilitate weight loss are also assessed post-interaction. The Pre-Interaction Questionnaire also includes 3 weight loss motivational assessments, in which the participants are asked to rate their confidence, importance, and readiness to change their weight-related lifestyle behaviors using a ruler ranging from 1 to 10, where 1 indicates “not at all” and 10 indicates “very much” [21]. These assessments are repeated in the Post-Interaction and Follow-Up Questionnaires. Completing this questionnaire should take no more than 15 minutes.

#### Post-Interaction Questionnaire

This instrument includes 16 five-point Likert-scale questions adapted from PCTQ-P and the confidence, importance, and readiness rulers assessed in the Pre-Interaction and Follow-Up Questionnaires. Completing this questionnaire should take no more than 15 minutes.

#### Usability Questionnaire

This instrument includes 15 five-point Likert-scale questions from the Telehealth Usability Questionnaire [116], a validated measure that combines the questions from popular traditional computer technology questionnaires, such as the Technology Acceptance Model [117] and the IBM Post-Study System Usability Questionnaire [118]. The Telehealth Usability Questionnaire was adapted for this study to assess perceptions of NAOMI’s usability, such as usefulness, ease of use, reliability, and satisfaction. Completing this questionnaire should take no more than 15 minutes.

#### Consultation and Relational Empathy Measure

The 10-question validated Consultation and Relational Empathy (CARE) measure [119] was adopted by the investigators to assess NAOMI’s empathic skills. Answers are converted to a numeric summary score on a 0-100 scale allowing the investigators to directly compare NAOMI’s empathic skills with those of human or other AI counselors. Completing this measure should take no more than 10 minutes.

#### Follow-Up Questionnaire

This instrument includes the confidence, importance, and readiness rulers assessed in the Pre- and Post-Interaction Questionnaires. Completing this questionnaire should take no more than 5 minutes.

#### Semi-Structured Qualitative Interview

Research staff will use a semi-structured interview guide composed of 13 questions to query participants’ prior experience with other conversational agents or AI counselors and to gather feedback on their interaction with NAOMI, including the usefulness of NAOMI’s advice, quality of its counseling, and suggestions for improving NAOMI. The qualitative interview should take no more than 45 minutes.

### Recruitment

#### Setting

We will recruit participants from the primary care clinics in Detroit, Michigan, and the greater Metropolitan Detroit area that are part of the MetroNet primary care practice-based research network [120]. Developed in 2001 with federal funding, MetroNet is a collaborative effort of the Wayne State University’s Department of Family Medicine and Public Health Sciences, its affiliated family medicine residency programs, and 10 community-based primary care practices serving the diverse population of approximately 50,000 patients from Metropolitan Detroit (≈60% identifying as African American, 30% White, 5% Arab/Chaldean, and 5% Hispanics).

#### Inclusion Criteria and Target Sample Size

To be included in this study, patients must be (1) English-speaking, (2) aged 18 years 0 months to 65 years 0 months, and (3) meet criteria for overweight or obesity (BMI 25.0-39.9) as documented by their treating physician. The exclusion criteria are in line with other similar studies [121]: (1) comorbid conditions that might compromise data integrity or the patient’s ability to autonomously interact with NAOMI (eg, severe autism, schizophrenia, or other psychotic disorders), (2) patients whose elevated weight is secondary to another medical condition (eg, Prader-Willi), and (3) women who are pregnant. Following literature recommendations for sample size in feasibility studies [122,123], we are planning to recruit 50 (12-13 per each of the 4 study cycles to account for possible attrition between the study visit and a follow-up survey) participants to ultimately have 40 participants (10 per cycle), who will have completed both the study visit and a follow-up survey.

#### Procedures

MetroNet primary care clinics will be sent an introductory email describing this study and inviting them to serve as recruitment partners. Emails will be followed up by the study staff, who will make phone calls to assess interest and discuss the project in more detail. Clinics that agree to partner will be asked to identify a staff member to liaise with the study team in the recruitment of study participants from their clinical population. Patients eligible for participation in this study will be identified through a review of the participating clinics’ medical charts either by clinic or authorized study staff and recruited using 2 approaches.

In the first approach, clinicians will briefly introduce the study to eligible patients at the end of their in-person clinic visit. If a patient is interested in learning more about the study, they will either sign a Release of Contact Information Form to be forwarded to the study staff for follow-up or, if study staff are available in the clinic, they will meet with the patient to discuss the study and their interest in participating.

In the second approach, all potentially eligible patients identified via chart review will be mailed a letter of introduction explaining the purpose of the study and offering the opportunity to opt out of further contact. The study staff will follow up with the patients who do not opt out to present the details of the study, assess their interest in participating, and schedule interested patients for their study visit.

### System Versions and Training

We will develop and evaluate NAOMI in 4 stages. To determine the general direction for developing NAOMI, in stages 1 and 2, it will be implemented using an open-source foundation LLM and (1) using few-shot learning based on a prompt with task-specific instructions and examples of key counselor communication behaviors, and (2) using a domain adaptation strategy based on 1555 transcripts of psychotherapy sessions and 205 transcripts of MI sessions gathered in our previously funded studies to fine-tune the LLM with communication patterns and the language of MI counseling. In stages 3 and 4, we will refine the best approach.

### Data Analysis

We will use a combination of statistical, qualitative, and thematic approaches to analyze the data collected during this study.

#### Statistical Analysis

NAOMI’s adherence to the core principles of MI will be assessed using the MITI coding scheme [111], a standard instrument for assessing MI fidelity. Two trained MITI coders (the primary and secondary) will code NAOMI’s utterances in the transcripts of its interactions with the study participants with MITI codes. The primary coder will code all transcripts with the second coder co-coding randomly selected 25% of transcripts to assess interrater agreement using the Krippendorff α statistic [124]. The quantitative assessment of NAOMI’s counseling skills based on the MITI-coded transcripts has 2 components: global scores and behavior counts. The global scores aim to characterize the overall quality of patient-counselor interaction and include 4 dimensions (Cultivating Change Talk, Softening Sustain Talk, Partnership, and Empathy). Behavior counts are evaluated by tallying instances of specific counselor behaviors, which can be grouped into 5 broad categories: questions, reflections, MI adherent behavior, MI nonadherent behavior, and neutral behaviors. The global scores and behavior counts will then be used to assess the level of NAOMI’s counseling skills according to the MITI manual.

#### Qualitative Analysis

Due to the small sample size, only qualitative analysis of changes in the measures of perceptions of AI agents and counselors (administered in the Pre- and Post-Interaction Questionnaires) and motivational precursors (administered in Pre-, Post-Interaction, and Follow-Up Questionnaires) will be performed.

#### Thematic Analysis

Framework matrix analysis [125], an efficient, systematic approach to thematic analysis that begins with the construction of a matrix based on content areas derived from the interview guide, will be used to analyze transcribed qualitative interviews. Two coders will independently code the interviews by “charting” a summary of feedback into the matrix. Coders will meet to review and compare their matrices, resolve discrepancies, and develop a final coded-to-consensus matrix. Coders then identify emergent themes to summarize users’ experience with NAOMI and suggestions for intervention modification.

### Ethical Considerations

This research protocol was reviewed and approved by the institutional review board of Wayne State University (protocol IRB-23-01-5404), as amended, on May 25, 2024. After completing the study visit, the participants will be compensated with a US $75 Amazon gift card. A US $15 Amazon gift card will be provided to the participants after completing the Follow-Up Questionnaire. The data set for fine-tuning the LLM underlying NAOMI is fully deidentified. Transcripts of participants’ interactions with NAOMI will be modified by the study staff after the interaction to remove any identifying information. Audio and video data collected during qualitative interviews will be transcribed and deidentified. After transcription has been completed and the integrity of the data confirmed, the audio and video files will be destroyed. All survey data will be collected using a unique research identification number, which will also be used as a link to the participant’s survey data and to label the data derived from the participant’s interaction with NAOMI. The linkage between the research identification number and the participant’s identity will be maintained in a password-protected file stored on a secure server.

## Results

The first version of NAOMI has been developed. The investigative team also developed a website for the study visit, which will allow only study participants to access NAOMI, automatically guide the participants through the steps of the study visit detailed in Textbox 1, keep track of their progress, and allow them to complete the study visit only once. Participant recruitment will commence in September 2024. Data collection activities are expected to conclude in May 2025.

## Discussion

### Study Hypotheses

This study explores the hypotheses that NAOMI, an LLM-based AI counselor, can conduct unscripted weight loss MI sessions achieving: (1) at least intermediate MI fidelity; (2) 80% user satisfaction, and (3) positively shifting motivational precursors. LLM-based counselors have the potential to address the following limitations of scripted counselors.

First, LLMs can generate fluid, diverse, and knowledgeable AI agent responses that take into account previous interactions in a conversational session. Diversity (mixing informational statements, questions, reflections, and affirmations) and contextual relevance of counselor responses are integral parts of MI. Receiving short, generic, or repetitive replies from scripted and retrieval-based conversational agents for mental health care in response to deeply personal questions has been identified as one of the primary reasons for patient dissatisfaction in prior qualitative studies [87].

Second, LLMs possess human-level capabilities for generating fluent and coherent content. They recognize fine nuances of language and can be taught the concepts of style and tone. They can also pay close attention to patient language and communicate emotions and empathy. Understanding and paying attention to the nuances of patient language is necessary to generate engaging and contextually relevant responses.

Third, due to the open and loosely structured nature of MI counseling sessions, it is infeasible to capture all possible session contexts and scenarios with a finite set of rules, patterns, scripts, or dialog states. As part of this study, we will explore the feasibility of training LLMs to conduct MI counseling sessions by using context-appropriate provider communication skills to elicit target patient language and respond to client resistance.

### Limitations, Risks, and Mitigation Strategies

At the same time, LLMs (and consequently conversational agents based on them) have several known limitations and risks. First, LLMs are trained on large human-authored textual corpora and thus can learn the biases and stereotypes expressed in these corpora. Training corpora may also make a conversational agent personalized to the users in a specific region or belonging to a particular culture or demographic group and thus less relatable and effective for other users. To mitigate this risk, the LLM underlying NAOMI will be fine-tuned on a large and diverse corpus of psychotherapy and MI counseling session transcripts.

The “black box” nature of LLMs is another source of potential risk since it translates into minimal control over what and understanding of how LLM-based conversational agents generate their responses. LLMs are also known to be prone to “hallucinations” [126], that is, generating outputs that are fluent and plausible, yet factually incorrect. The lack of interpretability and controllability is, however, common to many machine learning models and scientific understanding of the mechanism by which LLMs generate their output is still in its infancy [127]. The risk of NAOMI generating inappropriate or factually incorrect content will be mitigated by a study team member observing NAOMI’s interaction with every study participant and intervening should the interaction go awry.

Since study participants in each recruitment wave are not randomized to different conditions, our results may also be subject to cohort effects. Once NAOMI is fully developed and validated, we will address these limitations by conducting a randomized controlled trial testing NAOMI’s efficacy.

### Conclusions

This study aims to explore the feasibility of developing NAOMI, an LLM-based Web app for fully automated MI-style weight loss counseling. If proven effective, LLM-based counseling agents have the potential to become a cost-effective approach for addressing the obesity epidemic at a public health level. They can also have a broad, transformative effect on the delivery of MI and other psychotherapeutic modalities helping to mitigate the key barriers to their widespread adoption and enabling just-in-time and on-demand counseling anywhere. Upon successful completion of this study, we will measure NAOMI’s efficacy in improving motivational precursors and actual lifestyle behaviors in a randomized clinical trial.

## Appendix

Multimedia Appendix 1 NIH peer-review report.

## Abbreviations

AI: artificial intelligence
CARE: Consultation and Relational Empathy
LLM: large language model
MI: motivational interviewing
MITI: Motivational Interviewing Treatment Integrity
NAOMI: Neural Agent for Obesity Motivational Interviewing
PCTQ-P: Perceptions of Computerized Therapy Questionnaire-Patient

## Data Availability

The data sets generated and analyzed during this study will be available from the corresponding author upon reasonable request.
