# Supplementary material for: Neural Conversational Agent for Weight Loss Counseling: Protocol for an Implementation and Feasibility Study
Source: JMIR Res Protoc. 2024 Sep 20;13:e60361. doi: 10.2196/60361 (PMC11452760; doi:10.2196/60361)
Supplement: Multimedia Appendix 1 [file resprot_v13i1e60361_app1.pdf]

SUMMARY STATEMENT

(Privileged Communication)

PROGRAM CONTACT:
Kristopher Bough
(301) 496-2604
kristopher.bough@nih.gov

Release Date: 10/28/2022
Revised Date: 10/28/2022

Application Number: 1 R21 NR020388-01A1
Formerly: 1R21NR020388-01

Principal Investigator

KOTOV, ALEXANDER

Applicant Organization: WAYNE STATE UNIVERSITY

Review Group: CIDH
Clinical Informatics and Digital Health Study Section

Meeting Date: 09/29/2022
Council: JAN 2023
Requested Start: 05/15/2023

RFA/PA: PA20-195
PCC: DTCKB
Dual IC(s): LM, HD

Project Title: Neural Conversational Agent for Automated Weight Loss Counseling

SRG Action: Impact Score:23
Next Steps: Visit https://grants.nih.gov/grants/next_steps.htm
Human Subjects: 30-Human subjects involved - Certified, no SRG concerns
Animal Subjects: 10-No live vertebrate animals involved for competing appl.
Gender: 1A-Both genders, scientifically acceptable
Minority: 1A-Minorities and non-minorities, scientifically acceptable
Age: 3A-No children included, scientifically acceptable

Project Year	Direct Costs Requested	Estimated Total Cost
1	150,000	220,091
2	125,000	183,409
TOTAL	275,000	403,500

ADMINISTRATIVE BUDGET NOTE: The budget shown is the requested budget and has not been adjusted to reflect any recommendations made by reviewers. If an award is planned, the costs will be calculated by Institute grants management staff based on the recommendations outlined below in the COMMITTEE BUDGET RECOMMENDATIONS section.

1R21NR020388-01A1 Kotov, Alexander

RESUME AND SUMMARY OF DISCUSSION: This resubmitted R21 seeks to develop and assess the feasibility and usability of an automated, artificial intelligence driven agent to perform motivational interviewing (MI) for the purposes of providing weight loss behavior change counseling. The review panel found the application to be of high significance for reducing the barriers to adopting healthy behavior change. The application was expected to have a high impact on public health for its potential for use at scale and for overcoming limitations of patients to access to MI counseling. The panel noted that they would have like to see more refined discussion in the rigor of prior research of the need for improvements in particular racial and ethnic groups that may particularly benefit from such an approach. The investigators were noted for their deep expertise in computer science, motivational interviewing, and behavior change, bioinformatics, and obesity. Their history of prior collaboration was viewed as less clearly laid out. The application's technological innovations, particularly its use of neural networks AI for motivational interviewing was highlighted as an innovation of the application. The review panel found the approach as strong, noting that Aim 2's mixed methods feasibility and usability assessment for iterative improvement of the automated MI as a particular strength. Two issues raised in the presentation was the perception of a lack of Latino representation in the enrollment, and insufficient consideration of likely variable feasibility results based on literacy. The resubmission was viewed as very responsive, addressing issues that significantly strengthened the proposed research. The panel assessed the environment as outstanding, with strong computational resources and access to recruitment opportunities for the targeted clinical population. In the panel discussion, an issue that emerged that impacted how some panelist assessed the application was the strength of the review of prior research in providing evidence that MI was an effective intervention for weight loss, as well as a rigorous assessment of other digital solutions for weight loss management, as there are many. In the end, while some weighed the limited number of weaknesses to a greater degree, the overwhelming majority of the panel found the research would have a high impact for developing a novel AI approach to motivational intervening that may effectively lead to improved behavior change and outcomes for obese populations.

DESCRIPTION (provided by applicant): Obesity is one of the most important medical and public health problems in the United States. According to recent nationally representative studies, every third adult in the U.S. is obese. Motivational Interviewing (MI), a client-centered and directive approach to behavior change counseling, has been widely adapted for treating obesity. Despite the evidence presented in published meta-reviews that suggests that MI is effective at activating behavioral changes, anthropometric changes are less significant. At the same time, there are several access barriers to this type of behavioral health care, such as shortage of human counselors in certain geographical areas, long wait times, cost, and fear of judgment. Recent advances in deep learning have allowed artificial intelligence (AI) methods to expand into the areas of health care that were previously thought to be the exclusive province of human experts, such as clinical diagnostics. Behavioral health and MI, however, are the areas of medicine that have not yet substantially benefitted from modern AI technologies, such as neural conversational agents. To address this limitation, the proposed project aims to test the feasibility and usability of using neural conversational agents for automated behavioral counseling with a focus on weight loss. Specifically, we build on recent advances in deep learning, such as conversational agents, neural attention, transformers, supervised policy learning, variational autoencoders and adversarial training, and aim to develop and validate Neural Agent for Obesity Motivational Interviewing (NAOMI), a mobile device (smartphone or tablet) application to conduct automated MI counseling focused on weight loss. NAOMI is based on a novel neural architecture, which consists of neural networks that can be independently and collectively trained using the proposed multi-stage procedure to learn communication behaviors, which should be strategically utilized during different stages of an MI counseling session depending on the observed interactions and generate responses that are grounded in session context and reflect patient's language. We will recruit 40 obese adults, who will interact with NAOMI and provide their feedback through semi-structured qualitative

interviews. We plan on conducting at most 4 iterative development cycles of NAOMI with 10 patients participating in each cycle. We will conduct a mixed-methods sub-study after each development cycle. Quantitative evaluation of NAOMI's MI counseling skills will be conducted based on the transcripts of participants' interactions by a coder trained in using the MI Treatment Integrity (MITI) coding system, a standard instrument for assessing MI fidelity. Qualitative interviews with the participants will be analyzed using Framework Matrix Analysis. The methods and techniques proposed in this project can be adapted to other types of psychotherapeutic interventions besides MI and to other conditions besides obesity.

PUBLIC HEALTH RELEVANCE: The proposed research project is a feasibility and usability study of utilizing cutting-edge computer science methods – conversational agents, neural attention, transformers, supervised policy learning, variational autoencoders, and adversarial training – to develop an artificially intelligent agent for automated motivational interviewing counseling with a focus on weight loss. The proposed agent will circumvent barriers to healthcare providers' adoption of motivational interviewing, lack of fidelity to MI theory by human counselors, mitigate the issue of limited access to counseling services and complement human counselors in the behavioral healthcare delivery model.

CRITIQUE 1

Significance: 2
Investigator(s): 2
Innovation: 2
Approach: 3
Environment: 2

Overall Impact: This is a R21 resubmission from a mid-level faculty member with deep expertise in computer science. The goal of the proposal is to develop and then pilot test a conversational agent that can deliver motivational interviewing related to obesity. This is an important topic given the need to treat and prevent obesity for a growing population, and the need for digital tools to scale our outreach related to this important public health problem. The investigators were very responsive to the previous critiques, especially with expanding the participant audience of focus, the co-investigative team, and improving the feasibility of Aim 3. The expertise of the investigators is very strong and closely tied to the scientific goals of this proposal (i.e., representing both computer science from the PI as well as research expertise with motivational interviewing as well as behavioral science), and the environment for both computer science and patient recruitment is solid. Furthermore, the application is very innovative in its thoughtful consideration of methods (particularly neural architecture for conversational agents) specifically needed for broad conversations such as motivational interviewing that are less "task/decision" based. Finally, the methods involved in project are clearly articulated, for both the computational methods and design as well as the patient testing and piloting. There are a few minor weaknesses in the Approach of the proposal, such as the lack of clarity about other key patient-reported outcomes of this work and the need to clarify how the tool will address structural and social determinants of health, but these are minor in comparison to the overall potential of this proposal.

1. Significance:

Strengths

- Very clear description of the previous literature in the field, including the types of chatbots previously developed and the opportunity for motivational interviewing to make an impact on obesity outcomes

- Very strong rationale for focusing on digital health tools to scale health education support via motivational interviewing to more individuals

Weaknesses

- A clear theoretical framework could strengthen this application, such as how this digital platform deals with both process and content related to motivational interviewing for obesity
- More specific attention on structural and social determinants of obesity, as wel would improve the relevance of the digital health platform with respect to reducing inequities

2. Investigator(s):

Strengths

- PI has expertise in computer science and specifically machine learning relevant for creating novel conversational agents for motivational interviewing
- Co-I has clinical expertise relevant for obesity-related content

Weaknesses

- None

3. Innovation:

Strengths

- Use of neural based machine learning for the broad goals of motivational interviewing is very novel and promising

Weaknesses

- Still a bit of lack of clarity about whether content can be adapted based on age, gender, race/ethnicity, etc.

4. Approach:

Strengths

- Strong architectural depiction of how the machine learning will be created
- Clear explanation of how the tool will be trained and in iterative steps throughout the study
- Participant recruitment plan from existing clinical practices is well articulated

Weaknesses

- Statistical analysis and power calculation for understanding the reliability of chatbot-delivered motivational interviewing vs. “clinician gold standard” could be better articulated
- There are likely many secondary outcomes beyond “gold standard” motivational interviewing that are important to highlight within this work and may deserve more attention within the analysis plan, such as acceptability to patients, usage of the platform, etc.
- A bit more detail on the future ability to determine effectiveness of chatbot for motivational interviewing (such as in a future trial) could increase the strength of this application

5. Environment:

Strengths

- Strong informatics environment
- Existing computational infrastructure to create the final platform
- Existing clinical partner to facilitate participant recruitment

Weaknesses

- None

Study Timeline:

Strengths

- Timeline is presented broken down by quarters within the study period

Weaknesses

- None

Protections for Human Subjects:

Acceptable Risks and/or Adequate Protections

- Clear explanation of the potential risks and plans to mitigate risks are presented

Data and Safety Monitoring Plan (Applicable for Clinical Trials Only):

Not Applicable (No Clinical Trials)

Inclusion Plans:

- Sex/Gender: Distribution justified scientifically
- Race/Ethnicity: Distribution justified scientifically
- For NIH-Defined Phase III trials, Plans for valid design and analysis:
- Inclusion/Exclusion Based on Age: Distribution justified scientifically
    - The investigator described the plans for recruiting a diverse sample, but specific attention to Hispanic/Latino populations is needed.

Vertebrate Animals:

Not Applicable (No Vertebrate Animals)

Biohazards:

Not Applicable (No Biohazards)

Resource Sharing Plans:

Not Applicable (No Relevant Resources)

Authentication of Key Biological and/or Chemical Resources:

Not Applicable (No Relevant Resources)

Budget and Period of Support:

Recommend as Requested

CRITIQUE 2

Significance: 2
Investigator(s): 1
Innovation: 1
Approach: 2
Environment: 1

Overall Impact: This is a resubmission application proposing to use mixed methods to test the feasibility and usability of a Neural Agent for Obesity Motivational Interviewing, a mobile device application to conduct automated MI counseling focused on weight loss and assess the feasibility of utilizing NAOMI to conduct unscripted MI counseling with real patients. This study has the potential to be very impactful by using a neural conversational agent for MI and is novel in the area of behavioral counseling. The resubmission addressed 10 areas of concern, which has significantly strengthened the application and enthusiasm about the proposal. Specifically, positive score-driving factors included focusing on neural agent development and patient feedback using qualitative interviews; adding a clinical psychologist who specializes in behavioral medicine and obesity practice-based research methods; adding measurable success criteria for the second aim to inform if there's any scientific merit in moving forward. Non-score driving concerns that were weighted in the overall impact included that the researchers should: 1) ascertain the underlying reason for obesity (SDOH, mental health – depression/anxiety, etc). MI can affect behavior but intervention may not be sustainable if the underlying issue is not addressed; 2) assess SOGIE (sexual orientation gender identity and expression) demographics of the sample; 3) ensure the study staff conducting the home visits are persons of color or persons who have lived experience or expertise in these populations; 4) consider if the agent response generator should understand, be trained, or use modern vernacular? 5) address why are no Latinx persons anticipated on planned enrollment? Despite these concerns, the overall impact of this study is high and NAOMI can make a significant contribution to the field and possibly in other conditions where MI is appropriate.

1. Significance:

Strengths

- More than one-third of adults (36%) and one-fifth (20.5%) of adolescents aged 12-19 in the U.S. were obese.
- MI may be an efficient and cost-effective mechanism to deliver weight loss interventions using a neural agent.
- Previous study My-SCOPE foundational to proposed AI communication patterns and discriminator network – this study demonstrated feasibility of training the deep neural network for modeling and analyzing MI sessions.
- Accessibility of NAOMI on a smartphone or tablet.

Weaknesses

- No mention of specific demographics (race/ethnicity) of targeted sample or descriptive statistics illustrating why this is an urgent issue in proposed sample.

- No mention if MI has been successful in proposed study sample.

2. Investigator(s):

Strengths

- Dr. Kotov – has published and collaborated with Dr. Carcone. Expertise in information retrieval, neural language processing and biomedical informatics researcher with extensive expertise in modeling, analyzing and searching textual data.
- Dr. Carcone – Expertise on examining the mechanisms of effect in Motivational Interviewing, development and testing of behavioral interventions to improve health outcomes with an emphasis on pediatric and emerging adults with chronic illnesses, including obesity.
- Dr. Towner – Expertise in developing, testing, and implementing family-based health behavioral interventions to reduce obesity in families from low-income and minority backgrounds in clinical, community, and home settings.

Weaknesses

- PI and Dr. Towner have not published together.

3. Innovation:

Strengths

- Technical innovation - Using neural AI agent for motivational interviewing is fundamentally different from all other types of dialog systems.
- Limited prior research on neural AI agents for motivational interviewing.

Weaknesses

- None.

4. Approach:

Strengths

- Motivational Interviewing is an empirically supported strategy for enhancing intrinsic motivation for behavior change.
- Leverages mixed methods (MITI coding instrument and semi-structured interviews) to address specific aims.
- Using previously tested and standardized recruitment procedures based on researchers past clinical trials.
- Described proposed strategies for increasing fidelity in data collection.

Weaknesses

- No Latino persons anticipated on planned enrollment.
- Did not mention to collect SOGIE data from the investigator developed demographic form.
- No mention if study staff conducting home visits will be representative of the sample demographic. Also, did not clearly describe how they will receive training to engage in a non-judgmental manner – why would the study staff be in judgement?

5. Environment:

Strengths

- National research university with an urban teaching and service mission.
- Strong computer science infrastructure.
- Collaboration with family medicine and public health sciences.

Weaknesses

- None.

Protections for Human Subjects:

Acceptable Risks and/or Adequate Protections

Data and Safety Monitoring Plan (Applicable for Clinical Trials Only):

Acceptable

Inclusion Plans:

- Sex/Gender: Distribution justified scientifically
- Race/Ethnicity: Distribution justified scientifically
- For NIH-Defined Phase III trials, Plans for valid design and analysis: Not applicable
- Inclusion/Exclusion Based on Age: Distribution justified scientifically
  - No Latinos included on anticipated enrollment.

Vertebrate Animals:

Not Applicable (No Vertebrate Animals)

Biohazards:

Not Applicable (No Biohazards)

Revision:

- The resubmission addressed 10 areas of concern, which has significantly strengthened the application and enthusiasm about the proposal. Specifically, positive score-driving factors included focusing on neural agent development and patient feedback using qualitative interviews; adding a clinical psychologist who specializes in behavioral medicine and obesity practice-based research methods; adding measurable success criteria for the second aim to inform if there's any scientific merit in moving forward.

Resource Sharing Plans:

Not Applicable (No Relevant Resources)

Authentication of Key Biological and/or Chemical Resources:

Not Applicable (No Relevant Resources)

Budget and Period of Support:

Recommend as Requested

CRITIQUE 3

Significance: 2
Investigator(s): 1
Innovation: 1
Approach: 3
Environment: 1

Overall Impact: This application is a resubmission, and it proposes a feasibility study to use neural conversational agents to deliver motivational interviewing (MI) counseling to adults with obesity. The study is significant and novel to develop computerized MI interventions for weight loss interventions. Additional strengths of the application include the strong investigative team, excellent research environment, preliminary data from a prototype conversational agent, and a sound plan for the refinement of system architecture. There are some moderate weaknesses. For instance, since the last submission to this submission, the rationale for switching the study population from adolescents (initial submission) to adults (18-65, this resubmission) is not well justified scientifically. Whether the feasibility of the neural conversational agents-based motivational interviewing (MI) counseling is varied across subpopulations (e.g., literacy, age, and sex) is not considered. Overall, the study's strengths outweigh its limitations. The application is judged to have a high impact.

1. Significance:

Strengths

- Obesity is a major public health concern and is highly prevalent.
- People face major challenges in accessing obesity-related interventions, such as access to specialists, stigma, and self-motivation.
- If successful, the study will have a high public health impact that develops a neural conversational agent for motivating weight loss.

Weaknesses

- None noted by Reviewer

2. Investigator(s):

Strengths

- The study team is strong and has the necessary expertise to carry out the study.

Weaknesses

- None noted by Reviewer

3. Innovation:

Strengths

- The study is conceptually novel to develop an AI conversational agent for motivating weight loss.
- The proposed AI conversational agent is methodological novel in several fundamental ways including the category of the dialog system.

Weaknesses

- None noted by Reviewer

4. Approach:

Strengths

- The preliminary data has shown the feasibility that AI conversational agents can mimic some aspects of MI counseling
- The modular neural architecture is well stated with sufficient details.
- The recruitment is well planned and sounds feasible

Weaknesses

- Since the last submission to this submission, the rationale for switching the study population from adolescents (initial submission) to adults (18-65, this resubmission) is not well justified scientifically
- Whether the feasibility of the neural conversational agents-based motivational interviewing (MI) counseling is varied across subpopulations (e.g., literacy, age, and sex) is unclear.

5. Environment:

Strengths

- The environment at Wayne State University is excellent to conduct the proposed study, the research infrastructure is strong to support the project.

Weaknesses

- None noted by Reviewer

Study Timeline:

Strengths

- None noted by Reviewer

Weaknesses

- None noted by Reviewer

Protections for Human Subjects:

Acceptable Risks and/or Adequate Protections

- Protections for human subjects plans are appropriate.

Data and Safety Monitoring Plan (Applicable for Clinical Trials Only):

Not Applicable (No Clinical Trials)

Inclusion Plans:

- Sex/Gender: Distribution justified scientifically
- Race/Ethnicity: Distribution justified scientifically
- For NIH-Defined Phase III trials, Plans for valid design and analysis: Not applicable
- Inclusion/Exclusion Based on Age: Distribution justified scientifically

Vertebrate Animals:
Not Applicable (No Vertebrate Animals)

Biohazards:
Not Applicable (No Biohazards)

Resubmission:

- The review comments from the first submission were properly addressed

Resource Sharing Plans:
Not Applicable (No Relevant Resources)

Authentication of Key Biological and/or Chemical Resources:
Not Applicable (No Relevant Resources)

Budget and Period of Support:
Recommend as Requested

THE FOLLOWING SECTIONS WERE PREPARED BY THE SCIENTIFIC REVIEW OFFICER TO SUMMARIZE THE OUTCOME OF DISCUSSIONS OF THE REVIEW COMMITTEE, OR REVIEWERS' WRITTEN CRITIQUES, ON THE FOLLOWING ISSUES:

PROTECTION OF HUMAN SUBJECTS: ACCEPTABLE

INCLUSION OF WOMEN PLAN: ACCEPTABLE

INCLUSION OF MINORITIES PLAN: ACCEPTABLE

INCLUSION ACROSS THE LIFESPAN: ACCEPTABLE

COMMITTEE BUDGET RECOMMENDATIONS: The budget was recommended as requested.

Footnotes for 1 R21 NR020388-01A1; PI Name: Kotov, Alexander

NIH has modified its policy regarding the receipt of resubmissions (amended applications).See Guide Notice NOT-OD-18-197 at https://grants.nih.gov/grants/guide/notice-files/NOT-OD-18-197.html. The impact/priority score is calculated after discussion of an application by averaging the overall scores (1-9) given by all voting reviewers on the committee and multiplying by 10. The criterion scores are submitted prior to the meeting by the individual reviewers assigned to an application, and are not discussed specifically at the review meeting or calculated into the overall impact score. Some applications also receive a percentile ranking. For details on the review process, see http://grants.nih.gov/grants/peer_review_process.htm#scoring.

MEETING ROSTER

Clinical Informatics and Digital Health Study Section
Healthcare Delivery and Methodologies Integrated Review Group
CENTER FOR SCIENTIFIC REVIEW
CIDH
09/29/2022 - 09/30/2022

Notice of NIH Policy to All Applicants: Meeting rosters are provided for information purposes only. Applicant investigators and institutional officials must not communicate directly with study section members about an application before or after the review. Failure to observe this policy will create a serious breach of integrity in the peer review process, and may lead to actions outlined in NOT-OD-22-044 at https://grants.nih.gov/grants/guide/notice-files/NOT-OD-22-044.html, including removal of the application from immediate review.

CHAIRPERSON(S)

DEXHEIMER, JUDITH W, PHD
ASSOCIATE PROFESSOR
DEPARTMENT OF PEDIATRICS
AND BIOMEDICAL INFORMATICS
CINCINNATI CHILDREN'S HOSPITAL MEDICAL CENTER
CINCINNATI, OH 45229

MEMBERS

ABDULLAH, SAEED, PHD *
ASSISTANT PROFESSOR
COLLEGE OF INFORMATION SCIENCES AND TECHNOLOGY
PENNSYLVANIA STATE UNIVERSITY
UNIVERSITY PARK, PA 16802

ALPERN, ELIZABETH RACHEL, MD
PROFESSOR
DEPARTMENT OF PEDIATRICS
ANN AND ROBERT H. LURIE CHILDREN'S HOSPITAL
FEINBERG SCHOOL OF MEDICINE
NORTHWESTERN UNIVERSITY
CHICAGO, IL 60611

BARTLETT, CHRISTOPHER WILIAM, PHD *
ASSOCIATE PROFESSOR OF PEDIATRICS
DEPARTMENT OF PEDIATRICS/ BATTLE CENTER FOR
MATHEMATICAL MEDICINE
NATIONWIDE CHILDREN'S HOSPITAL
COLUMBUS, OH 43205

CHON, KI H, PHD
PROFESSOR
DEPARTMENT OF BIOMEDICAL ENGINEERING
UNIVERSITY OF CONNECTICUT
STORRS, CT 06269

CHUNG, JANE, PHD *
ASSISTANT PROFESSOR
SCHOOL OF NURSING
VIRGINIA COMMONWEALTH UNIVERSITY
RICHMOND, VA 23298

DIAS, ROGER DAGLIUS, PHD, MD, MBA *
ASSISTANT PROFESSOR, DIRECTOR OF RESEARCH AND
INNOVATION, STRATUS CENTER FOR MEDICAL
SIMULATION, DIRECTOR AND LEAD INVESTIGATOR, HUMAN
FACTORS AND COGNITIVE ENGINEERING LAB
DEPARTMENT OF EMERGENCY MEDICINE
HARVARD UNIVERSITY
BOSTON, MA 02115

ERICKSON, BRADLEY J, PHD *
PROFESSOR
DEPARTMENT OF RADIOLOGY
MAYO CLINIC
ROCHESTER, MN 55905

FENTON, SUSAN HRACHOVY, PHD, MBA *
ASSOCIATE PROFESSOR
SCHOOL OF BIOMEDICAL INFORMATICS
UNIVERSITY OF TEXAS HEALTH SCIENCE CENTER
HOUSTON, TX 77030

FRONTERA, JENNIFER ANN, MD *
PROFESSOR
DEPARTMENT OF NEUROLOGY
NEW YORK GROSSMAN SCHOOL OF MEDICINE
NEW YORK, NY 10016

GRUNDMEIER, ROBERT W, MD
DIRECTOR OF CLINICAL INFORMATICS
DEPARTMENT OF BIOMEDICAL AND HEALTH INFORMATICS
CHILDREN'S HOSPITAL OF PHILADELPHIA
PHILADELPHIA, PA 19146

GUO, JINGCHUAN, PHD *
ASSISTANT PROFESSOR
DEPARTMENT OF PHARMACEUTICAL OUTCOMES AND
POLICY
COLLEGE OF PHARMACY
UNIVERSITY OF FLORIDA
GAINESVILLE, FL 32610

HATEF-NAIMI, ELHAM, MD, MPH *
ASSISTANT PROFESSOR
CENTER FOR POPULATION HEALTH IT
DEPARTMENT OF HEALTH POLICY AND MANAGEMENT
SCHOOL OF PUBLIC HEALTH
JOHNS HOPKINS UNIVERSITY
BALTIMORE, MD 21205

JEFFERY, ALVIN DEAN, PHD, RN *
ASSISTANT PROFESSOR
DEPARTMENT OF BIOMEDICAL INFORMATICS
SCHOOL OF NURSING
VANDERBILT UNIVERSITY
NASHVILLE, TN 37240

JORDAN, JENNIFER HAWTHORNE, PHD *
ASSISTANT PROFESSOR
DEPARTMENT OF BIOMEDICAL ENGINEERING
AND PAULEY HEART CENTER
VIRGINIA COMMONWEALTH UNIVERSITY
RICHMOND, VA 23284

KAMALESWARAN, RISHIKESAN, PHD *
DIRECTOR OF TRANSLATIONAL CLINICAL INFORMATICS,
ASSISTANT PROFESSOR
DEPARTMENT OF BIOMEDICAL INFORMATICS
PEDIATRICS, AND EMERGENCY MEDICINE
EMORY UNIVERSITY SCHOOL OF MEDICINE
ATLANTA, GA 30322

KENT, DAVID M, MD, CM *
PROFESSOR OF MEDICINE, NEUROLOGY AND CLINICAL
AND TRANSLATIONAL SCIENCE, DIRECTOR, PREDICTIVE
ANALYTICS AND COMPARATIVE EFFECTIVENESS (PACE)
CENTER
PREDICTIVE ANALYTICS AND COMPARATIVE
EFFECTIVENESS CENTER
INSTITUTE FOR CLINICAL RESEARCH HEALTH POLICY
TUFTS MEDICAL CENTER
BOSTON, MA 02111

KOVELL, LARA, MD *
ASSISTANT PROFESSOR
DEPARTMENT OF MEDICINE
CHAN MEDICAL SCHOOL
UNIVERSITY OF MASSACHUSETTS
WORCESTER, MA 01655

LIU, FEIFAN, PHD *
ASSISTANT PROFESSOR
DEPARTMENT OF POPULATION
AND QUANTITATIVE HEALTH SCIENCES
CHAN MEDICAL SCHOOL
UNIVERSITY OF MASSACHUSETTS
WORCESTER, MA 01605

LYLES, COURTNEY REES, PHD
ASSOCIATE PROFESSOR
DEPARTMENT OF MEDICINE
UNIVERSITY OF CALIFORNIA, SAN FRANCISCO
SAN FRANCISCO, CA 94110

MAHAJAN, AMAN, PHD *
PETER AND EVA SAFAR PROFESSOR AND CHAIR,
PROFESSOR OF BIOENGINEERING AND BIOMEDICAL
INFORMATICS
DEPARTMENT OF ANESTHESIOLOGY AND
PERIOPERATIVE MEDICINE
UNIVERSITY OF PITTSBURGH
PITTSBURGH, PA 15261

MARQUINE, MARIA, PHD *
ASSOCIATE PROFESSOR
DEPARTMENT OF MEDICINE, GERIATRICS DIVISION
DUKE CENTER FOR THE STUDY OF AGING
AND HUMAN DEVELOPMENT
DUKE UNIVERSITY SCHOOL OF MEDICINE
DURHAM, NC 27710

POLLACK, ARI, MD, MS *
ASSOCIATE PROFESSOR, ADJUNCT ASSOCIATE
PROFESSOR INFORMATION SCHOOL,
DIVISION OF NEPHROLOGY
DEPARTMENT OF PEDIATRICS
DEPARTMENT OF BIOMEDICAL INFORMATICS AND MEDICAL
EDUCATION, UNIVERSITY OF WASHINGTON
SEATTLE, WA 98195-9472

PRESCOTT, HALLIE CHRISTINE, MD, MS
ASSOCIATE PROFESSOR
DEPARTMENT OF INTERNAL MEDICINE
UNIVERSITY OF MICHIGAN
ANN ARBOR, MI 48109

RAMOS, SILVIA RAQUEL, PHD, MBA *
ASSOCIATE PROFESSOR OF NURSING AND PUBLIC HEALTH
SCHOOL OF NURSING
DEPARTMENT OF SOCIAL AND BEHAVIORAL SCIENCES
SCHOOL OF PUBLIC HEALTH
YALE UNIVERSITY
NEW HAVEN, CT 06520

REHG, JAMES M, PHD
PROFESSOR
CENTER FOR BEHAVIORAL IMAGING
SCHOOL OF INTERACTIVE COMPUTING
GEORGIA INSTITUTE OF TECHNOLOGY
ATLANTA, GA 30332

RIOS, ANTHONY, PHD *
ASSISTANT PROFESSOR
DEPARTMENT OF INFORMATION SYSTEMS
AND CYBER SECURITY
CARLOS ALVAREZ COLLEGE OF BUSINESS
UNIVERSITY OF TEXAS SAN ANTONIO
SAN ANTONIO, TX 78249

SEO, NA JIN, PHD
PROFESSOR
DEPARTMENTS OF HEALTH PROFESSIONS AND
HEALTH SCIENCES AND RESEARCH
MEDICAL UNIVERSITY OF SOUTH CAROLINA
CHARLESTON, SC 29425

SHYU, CHI-REN, PHD *
DIRECTOR, INSTITUTE FOR DATA SCIENCE AND INFORMATICS PAUL K. AND DIANNE SHUMAKER PROFESSOR
COLLEGE OF ENGINEERING
UNIVERSITY OF MISSOURI
COLUMBIA, MO 65211

SONG, XUBO, PHD *
PROFESSOR
DEPARTMENT OF COMPUTER SCIENCE AND ELECTRICAL ENGINEERING
SCHOOL OF MEDICINE
OREGON HEALTH AND SCIENCE UNIVERSITY
PORTLAND, OR 97239

SPIEGEL, BRENNAN, MD, MPH *
PROFESSOR OF MEDICINE AND PUBLIC HEALTH
DIRECTOR OF HEALTH SERVICES RESEARCH
CEDARS-SINAI CEDARS-SINAI SITE DIRECTOR
CLINICAL AND TRANSLATIONAL SCIENCE INSTITUTE
LOS ANGELES, CA 90048

STAVRAKIS, STAVROS, PHD, MD *
ASSOCIATE PROFESSOR OF MEDICINE
DIRECTOR, CARDIOVASCULAR RESEARCH
UNIVERSITY OF OKLAHOMA HEALTH SCIENCES CENTER
OKLAHOMA CITY, OK 73104

SWARD, KATHERINE ANN, PHD, RN
PROFESSOR
DEPARTMENT OF BIOMEDICAL INFORMATICS
COLLEGE OF NURSING
UNIVERSITY OF UTAH
SALT LAKE CITY, UT 84112

TANDON, ANIMESH, MD, MS *
DIRECTOR OF CARDIOVASCULAR INNOVATION
CLINICAL INNOVATION LEAD, PEDIATRIC INSTITUTE
CLEVELAND CLINIC
CLEVELAND, OH 44195

TOPAZ, MAXIM, PHD, RN
ASSOCIATE PROFESSOR
SCHOOL OF NURSING
COLUMBIA UNIVERSITY
NEW YORK, NY 10032

WADE, ERIC, PHD
ASSOCIATE PROFESSOR
DEPARTMENT OF MECHANICAL, AEROSPACE AND BIOMEDICAL ENGINEERING
UNIVERSITY OF TENNESSEE, KNOXVILLE
KNOXVILLE, TN 37996

WEI, JEANNE Y, PHD, MD *
EXECUTIVE DIRECTOR, REYNOLDS INSTITUTE ON AGING
PROFESSOR AND CHAIRPERSON
REYNOLDS DEPARTMENT OF GERIATRICS
COLLEGE OF MEDICINE
UNIVERSITY OF ARKANSAS FOR MEDICAL SCIENCES
LITTLE ROCK, AR 72205

MAIL REVIEWER(S)

LAKSHMINARAYAN, KAMAKSHI, PHD, MBBS, MS
PROFESSOR
DEPARTMENT OF NEUROLOGY, MEDICAL SCHOOL
SCHOOL OF PUBLIC HEALTH
UNIVERSITY OF MINNESOTA
MINNEAPOLIS, MN 55454

SCIENTIFIC REVIEW OFFICER

HEWETT, PAUL, PHD
SCIENTIFIC REVIEW OFFICER
CENTER FOR SCIENTIFIC REVIEW
NATIONAL INSTITUTE OF HEALTH
BETHESDA, MD 20892

EXTRAMURAL SUPPORT ASSISTANT

NJOKU, PHILIP C
EXTRAMURAL SUPPORT ASSISTANT
DIVISION OF AIDS, BEHAVORIAL, POPULATION SCIENCES
NATIONAL INSTITUTES OF HEALTH
BETHESDA, MD 20892

* Temporary Member. For grant applications, temporary members may participate in the entire meeting or may review only selected applications as needed.

Consultants are required to absent themselves from the room during the review of any application if their presence would constitute or appear to constitute a conflict of interest.
